# alphaB-crystallin is a marker of aggressive breast cancer behavior but does not independently predict for patient outcome: a combined analysis of two randomized studies

**DOI:** 10.1186/1472-6890-14-28

**Published:** 2014-06-23

**Authors:** Triantafyllia Koletsa, Flora Stavridi, Mattheos Bobos, Ioannis Kostopoulos, Vassiliki Kotoula, Anastasia G Eleftheraki, Irene Konstantopoulou, Christos Papadimitriou, Anna Batistatou, Helen Gogas, Angelos Koutras, Dimosthenis V Skarlos, George Pentheroudakis, Ioannis Efstratiou, Dimitrios Pectasides, George Fountzilas

**Affiliations:** 1 Department of Pathology, Aristotle University of Thessaloniki School of Medicine, University Campus, 54124 Thessaloniki, Greece; 2 Third Department of Medical Oncology, “Hygeia” Hospital, Athens, Greece; 3 Laboratory of Molecular Oncology, Hellenic Foundation for Cancer Research, Aristotle University of Thessaloniki School of Medicine, Thessaloniki, Greece; 4 Section of Biostatistics, Hellenic Cooperative Oncology Group, Data Office, Athens, Greece; 5 Molecular Diagnostics Laboratory, IRRP, National Centre for Scientific Research NCSR Demokritos, Athens, Greece; 6 Department of Clinical Therapeutics, “Alexandra” Hospital, University of Athens School of Medicine, Athens, Greece; 7 Department of Pathology, Ioannina University Hospital, Ioannina, Greece; 8 First Department of Medicine, “Laiko” General Hospital, University of Athens, Medical School, Athens, Greece; 9 Department of Medicine, Division of Oncology, University Hospital, University of Patras Medical School, Patras, Greece; 10 Second Department of Medical Oncology, “Metropolitan” Hospital, Piraeus, Greece; 11 Department of Medical Oncology, Ioannina University Hospital, Ioannina, Greece; 12 Department of Pathology, “Papageorgiou” Hospital, Thessaloniki, Greece; 13 Second Department of Internal Medicine, Oncology Section, “Hippokration” Hospital, Athens, Greece; 14 Department of Medical Oncology, “Papageorgiou” Hospital, Aristotle University of Thessaloniki School of Medicine, Thessaloniki, Greece

**Keywords:** Breast cancer, AlphaB-crystallin, Triple-negative breast cancer, Basal core phenotype, *BRCA* gene mutations, Taxane-based therapy

## Abstract

**Background:**

alphaB-crystallin is a small heat shock protein that has recently been characterized as an oncoprotein correlating with the basal core phenotype and with negative prognostic factors in breast carcinomas. The purpose of this study was to evaluate alphaB-crystallin with respect to clinicopathological parameters and the outcome of patients with operable high-risk breast cancer.

**Methods:**

A total of 940 tumors were examined, derived from an equal number of patients who had participated in two randomized clinical trials (paclitaxel-containing regimen in 793 cases). Immunohistochemistry for ER, PgR, HER2, Ki67, CK5, CK14, CK17, EGFR, alphaB-crystallin, BRCA1 and p53 was performed. *BRCA1* mutation data were available in 89 cases.

**Results:**

alphaβ-crystallin was expressed in 170 cases (18.1%) and more frequently in triple-negative breast carcinomas (TNBC) (45% vs. 14.5% non-TNBC, p < 0.001). alphaB-crystallin protein expression was significantly associated with high Ki67 (Pearson chi-square test, p < 0.001), p53 (p = 0.002) and basal cytokeratin protein expression (p < 0.001), *BRCA1* mutations (p = 0.045) and negative ER (p < 0.001) and PgR (p < 0.001). Its overexpression, defined as >30% positive neoplastic cells, was associated with adverse overall survival (Wald’s p = 0.046). However, alphaB-crystallin was not an independent prognostic factor upon multivariate analysis. No interaction between taxane-based therapy and aβ-crystallin expression was observed.

**Conclusions:**

In operable high-risk breast cancer, alphaB-crystallin protein expression is associated with poor prognostic features indicating aggressive tumor behavior, but it does not seem to have an independent impact on patient survival or to interfere with taxane-based therapy.

**Trial registrations:**

ACTRN12611000506998 (HE10/97 trial) and ACTRN12609001036202 (HE10/00 trial).

## Background

Small heat shock proteins (sHsps) are molecular chaperones and are expressed in response to a wide variety of unfavorable physiological and environmental conditions, playing a cytoprotective role. Their importance is reflected by the conservation of the a-crystallin structure from bacteria to humans [1]. alphaB-crystallin is a member of this sHsps family, found primarily in the lens of the eye in addition to various non-lenticular tissues [2-5]. This protein enhances survival in response to cellular stress by inhibiting protein aggregation, reducing intracellular reactive oxygen species levels [6] and inhibiting programmed cell death [7]. Inhibition of apoptosis is achieved by disrupting the proteolytic activation of caspase-3 [8,9] and by preventing translocation of Bcl-2 family members to the mitochondria [10]. Up to date, the underlying molecular mechanisms that engender alphaB-crystallin overexpression are poorly understood, although its prognostic value in cancer is now becoming more obvious [7,11].

Proteomic studies suggest that alphaB-crystallin may contribute in cancer development [12]. alphaB-crystallin has been found in malignant diseases, such as gliomas, prostate carcinomas, renal cell carcinomas and breast carcinomas [13-15], while its expression has been associated with poor clinical outcome in breast, hepatocellular and head and neck carcinomas [7,11,16,17]. Several studies have suggested that alphaB-crystallin expression is correlated with high histological grade, metastatic potential, poor clinical outcome and chemotherapy resistance in breast carcinomas [7,16,18]. Moreover, it is more commonly expressed in basal-like breast carcinomas (BLBC) and it is thought to contribute to their aggressive phenotype [19].

BLBC has emerged as a distinct breast cancer subtype by gene profiling studies [20,21] and is associated with short overall and disease-free survival. BLBC express proteins characteristic of basal epithelial cells, including basal cytokeratins (CK5/6 and/or CK14 and/or CK17) and commonly other markers such as p53, p-cadherin, alphaB-crystallin, vimentin and EGFR [21-27]. The expression of basal markers identifies a distinct subgroup of triple-negative breast cancer (TNBC), representing almost 75% of cases [23]. Moreover, there are several studies that suggest a link between BLBC and *BRCA1* mutational status [25,26,28].

The limited data on alphaB-crystallin in breast cancer suggest that there is a pathogenic link between alphaB-crystallin expression and BLBC [19,29]. In this study, the expression of alphaB-crystallin was evaluated in a large cohort of two randomized trials in order to evaluate possible associations with conventional clinicopathological characteristics, including established prognostic factors, such as histological grade, molecular subtypes and metastatic lymph node infiltration, and to investigate whether alphaB-crystallin is an independent prognostic/predictive marker.

## Methods

### Patients and tissues

The HE10/97 trial [30] was a randomized phase III trial (ACTRN12611000506998) in patients with high-risk node-negative or intermediate/high-risk node-positive operable breast cancer, comparing four cycles of epirubicin (E) followed by four cycles of intensified CMF (E-CMF) with three cycles of E, followed by three cycles of paclitaxel (T, Taxol®, Bristol Myers-Squibb, Princeton, NJ) followed by three cycles of intensified CMF (E-T-CMF). All cycles were given every two weeks with G-CSF support. Dose intensity of all drugs in both treatment arms was identical, but cumulative doses and duration of chemotherapy period differed. Totally, 595 eligible patients entered the study in a period of 3.5 years (1997–2000).

The HE10/00 trial [31,32] was a randomized phase III trial (ACTRN12609001036202) in which patients were treated with E-T-CMF (exactly as in the HE10/97 trial) or with four cycles of epirubicin/paclitaxel (ET) combination (given on the same day) every three weeks followed by three cycles of intensified CMF every two weeks (ET-CMF). By study design, the cumulative doses and the chemotherapy duration were identical in the two arms but dose intensity of epirubicin and paclitaxel was double in the E-T-CMF arm. A total of 1086 eligible patients with node-positive operable breast cancer were accrued in a period of 5 years (2000–2005).

Treatment schedules for the two studies, baseline characteristics and clinical outcomes of both trials have already been described in detail [30-33]. Clinical protocols were approved by local regulatory authorities, while the present translational research protocol was approved by the Bioethics Committee of the Aristotle University of Thessaloniki, School of Medicine, under the general title “Molecular investigation of the predictive and/or prognostic role of important signal transduction pathways in breast cancer” (A7150/18-3-2008). All patients signed a study-specific written informed consent before randomization, which in addition to providing consent for the trial allowed the use of their biological material for future research purposes.

In total, 271 patients from the HE10/97 trial and 669 from the HE10/00 trial were included in this study, based on tissue availability. Primary tumor diameter, axillary nodal status and tumor grade were obtained from the pathology report in each case.

### TMA construction

Formalin-fixed paraffin-embedded (FFPE) tissue samples from patient tumors (paraffin blocks) were collected retrospectively in the first trial (HE10/97) and prospectively in the second (HE10/00). The present study was carried out on tissue microarrays (TMAs). Representative hematoxylin-eosin stained sections from the tissue blocks were reviewed by two experienced in breast cancer pathologists and the most representative tumor areas were marked for the construction of ΤΜΑ blocks with a manual arrayer (Model I, Beecher Instruments, San Prairie, WI). Each tumor was represented by 2 tissue cores, 1.5 mm in diameter, which were obtained from different and most representative areas of primary invasive or in some cases from synchronous axillary lymph node metastases and re-embedded in recipient paraffin blocks. TMAs also contained control cores from different tumors and non-neoplastic tissues [32]. In total, 51 TMAs were created. Cases not represented, damaged or inadequate on the TMA sections were re-cut from the original blocks, where tissue material was still available.

### Immunohistochemistry (IHC) for breast cancer subtyping

IHC for ER (clone 6 F11, Leica Biosystems, Newcastle Upon Tyne, UK), PgR (clone 1A6, Leica Biosystems), HER2 (pl, code A0485, Dako, Glostrup, DK), Ki67 (clone MIB1, Dako), CK5 (clone XM26, Leica Biosystems), CK14 (clone LL002, Leica Biosystems), CK17 (E31, Leica Biosystems) and EGFR (clone 31G7, Invitrogen, Carlsbad, CA) was performed on serial 2 micron thick sections, using the Bond Max and Bond III autostainers (Leica Microsystems, Wetzlar, Germany), as previously described [34]. IHC was performed centrally at the Laboratory of Molecular Oncology of the Hellenic Foundation for Cancer Research, Aristotle University of Thessaloniki School of Medicine. Vimentin (clone V9, Dako) and cytokeratin 8/18 (clone 5D3, Leica Biosystems) were used as immunoreactivity controls and for the identification of tumor cells. Tissue samples negative with the above two antibodies were excluded from the study. The evaluation of all IHC sections was done by experienced breast cancer pathologists, blinded to the patient clinical characteristics and survival data.

All tumors included in this study were classified based on ER, PgR, HER2, Ki67, CK5 and EGFR, as Luminal A (ER-positive and/or PgR-positive, HER2-negative and Ki67^low^), Luminal B (ER-positive and/or PgR-positive, HER2-negative and Ki67^high^), luminal-HER2 (ER-positive and/or PgR-positive and HER2-positive), HER2-enriched (ER-negative, PgR-negative, HER2-positive) and TNBC (ER-negative, PgR-negative, HER2-negative). Tumors were classified as Ki67^high^ when ≥14% of neoplastic cells were positive [35]. TNBC of the basal core phenotype (BCP) were also distinguished as CK5-positive and/or EGFR-positive.

### IHC for alphaB-crystallin, BRCA1 and p53 markers

The IHC method was performed using the Bond Max and Bond III autostainers (Leica Microsystems). The Mouse IgG1 monoclonal antibody, clone 1B6.1-3G4 (Stressgen Biotechnologies, San Diego, CA) was used for the detection of full-length alphaB-crystallin (1:200 dilution, 1 h incubation at room temperature). The MS110 antibody from Merck KGaA (Darmstadt, Germany) was used for BRCA1 detection (1:150 dilution, for 20 min), while p53 protein was detected with the DO-7 clone (Dako) at dilution 1:100, for 20 min. The antigen–antibody complex was visualized using DAB as a chromogen. Slides were counterstained with Mayer’s hematoxylin for 10 min (Leica), washed in water, dehydrated and mounted.

### IHC evaluation

ER, PgR, HER2, Ki67 and EGFR protein expression was evaluated according to the established or proposed criteria [35-38]. CK5, CK14 and CK17 expression was considered as negative (absence of staining) or positive (any cytoplasmic staining of tumor cells) [34]. For alphaB-crystallin the percentage of positive tumor cells and the intensity (mild, moderate, strong) were recorded in every case. The distribution of continuous positivity values revealed a natural cut-off at 30%. Tumors were considered negative, when no specific cytoplasmic staining was observed, weakly positive (<30% positive neoplastic cells) and strongly positive (≥30% positive neoplastic cells). In the latter category staining intensity was predominantly strong; therefore, intensity was not included in the statistical analysis. The above staining pattern was in accordance with Moyano’s previous report [7], who used a cutoff of ≥30% to evaluate low and high alphaB-crystallin expressing tumors. BRCA1 staining was evaluated by using the histological score (H-score) at a positivity cut-off of > 100 [39]. For p53, ≥10% nuclear staining of invasive cancer cells was considered positive [40]. IHC positivity criteria for all antibodies are shown in Table 1.

**Table 1:** Criteria of immunohistochemical evaluation

**Protein**	**Scoring system**	**Cut-off**	**Staining pattern**
ER	H-Score	≥1%	N
PgR	H-Score	≥1%	N
HER2	0-3	>10%	M
p53	SQ	>10%	N
Ki67	SQ	≥14%	N
EGFR	0-3	>1%	M
CK5	SQ	any	C
CK14	SQ	any	C
CK17	SQ	any	C
CK8/18	SQ	any	C
Vimentin	SQ	any	C
alphaB-crystallin	Neg, W, S	>30	C
BRCA1	H-Score	>100	N

H-Score, histoscore; N, nuclear; M, membranous; C, cytoplasmic; SQ, semi-quantitative; Neg, negative; W, weakly positive; S, strongly positive.

HER2 status was also investigated in all cases with FISH using the ZytoLightH SPEC *HER2*/*TOP2A*/CEN17 triple color probe (ZytoVision, Bremerhaven, Germany), as previously described [41].

### BRCA mutations

DNA for *BRCA* screening was available for 127 of the 940 patients included in the present analysis. Seventy-nine patients were screened for *BRCA1* only, 3 patients were screened for *BRCA2* only and 7 patients were screened in both genes. Thirty-eight patients were not screened due to low quality of the DNA sample. Genomic DNA was isolated from peripheral blood lymphocytes following the salt extraction procedure [42]. The quantity and quality of the DNA samples were determined by UV absorbance using a Nanodrop™ 1000 (Thermo Fisher Scientific, MA) and agarose gel electrophoresis. *BRCA1* and *BRCA2* were amplified using intronic primer pairs flanking each exon and three diagnostic PCR reactions were also performed in order to detect the Greek founder genomic rearrangements involving exons 20, 23 and 24 [43]. PCR amplifications were performed in a Veriti 96-Well Thermal Cycler and the PCR products were directly sequenced using the v.3.1 BigDye Terminator Cycle Sequencing kit on an 3130XL Genetic Analyzer (all three from Applied Biosystems, Foster City, CA), according to the manufacturer’s instructions. In some cases of high-risk families, genomic DNA was also examined by MLPA analysis (MRC-Holland). Sequence variations, except well-known polymorphisms, were confirmed in an independent blood sample by sequencing both forward and reverse directions. All nucleotide numbers refer to the wild-type genomic DNA sequence of *BRCA1* NG_005905.2 and *BRCA2* NG_012772.1, as reported in RefSeqGene records. Primer sequences and protocols are available upon request.

### Statistical analysis

Categorical data are displayed as frequencies and corresponding percentages, while continuous data by median and range. Comparisons of categorical data between groups were performed by Fisher’s exact or Pearson chi-square tests. For continuous data, the assumptions for performing parametric tests were not fulfilled (the data were not normally distributed); therefore the non-parametric Kruskal-Wallis test was used. For numerical ordinal data, the Jonckheere-Terpstra trend test (JT test) was performed. Disease-free survival (DFS) was measured from the date of randomization until tumor recurrence, secondary neoplasm or death from any cause. Overall survival (OS) was measured from the date of randomization until death from any cause. Surviving patients were censored at the date of last contact. Time-to-event distributions were presented using Kaplan-Meier curves and compared using the log-rank test.

Univariate Cox regression analyses were performed for OS and DFS, to assess the prognostic or predictive significance in paclitaxel treatment of the examined biomarkers. A backward selection procedure with a removal criterion of p > 0.10 was performed in the multivariate Cox regression analysis in order to identify significant factors among the following: randomization group (ET-CMF, E-CMF, vs. E-C-MF), involved axillary lymph nodes (≥4 vs. 0–3), tumor grade (III-Undifferentiated vs. I-II), tumor size (>5 cm, 2–5 cm vs. ≤2 cm), type of surgery (breast conserving surgery vs. modified radical mastectomy, ΜRΜ), histological type (ductal vs. other) and adjuvant hormonal therapy (yes, missing vs. no). The examined markers were included in the final model using the categorization: alphaB-crystallin (strong positive, weak positive vs. negative), p53 (positive vs. negative) and BRCA1 (positive vs. negative).

Results of this study were presented according to reported recommendations for tumor marker prognostic studies [44]. The design of the study is prospective-retrospective, as previously described by Simon et al. [45]. All statistical tests were two sided and p < 0.05 was considered statistically significant. No adjustments for multiple tests are reported. The statistical analysis was conducted using the following statistical software: SPSS for Windows (version 15.0, IBM Corporation, NY) and SAS (version 9.3, SAS Institute Inc., Cary, NC).

## Results

### Clinicopathological characteristics of patients and tumor subtyping

A total of 940 patients with available FFPE tumor tissue blocks and successful assessment of alphaB-crystallin were included in the analysis. Selected patient and tumor characteristics are presented in Table 2. The majority of the patients were postmenopausal (54%) and underwent modified radical mastectomy (69%). The most common histological type was infiltrative ductal carcinoma, which accounted for 77.3% of the cases. Half of the tumors were of high histological grade and about 70% measured >2 cm. Almost two thirds of patients had 4 or more metastatic lymph nodes at the time of diagnosis. By using IHC for molecular subtyping [32], 24.3% of the tumors were classified as Luminal A, 39.5% as Luminal B, 13.8% as Luminal-HER2, 10.6% as HER2-enriched and 11.8% as TNBC.

**Table 2:** Selected patient and tumor characteristics

	**Study population**
	**N = 940**
	**N (%)**
Randomization arm	
E-T-CMF	454 (48.3)
E-CMF	147 (15.6)
ET-CMF	339 (36.1)
Age	
<50	381 (40.5)
≥50	559 (59.5)
Menopausal status	
Premenopausal	435 (46.3)
Postmenopausal	505 (53.7)
Type of surgery	
MRM	644 (68.5)
Breast conserving	296 (31.5)
Tumor size (cm)	
≤2	288 (30.6)
2,1-5	530 (56.4)
>5	122 (13.0)
Histological type	
Ductal	727 (77.3)
Lobular	97 (10.3)
Mixed	72 (7.7)
Other	44 (4.7)
N of positive nodes	
0-3	363 (38.6)
≥4	577 (61.4)
Histological grade	
I-II	470 (50.0)
III-Undifferentiated	470 (50.0)
Adjuvant HT	
No	178 (18.9)
Yes	739 (78.6)
Missing data	23 (2.4)
Adjuvant RT	
No	198 (21.1)
Yes	711 (75.6)
Missing data	31 (3.3)

N, number; E, epirubicin; T, paxlitaxel; C, cyclophosphamide; M, methotrexate; F, 5-FU; MRM, modified radical mastectomy; HT, hormonal therapy; RT, radiotherapy.

### alphaB-crystallin detection by IHC

**Figure 1:**
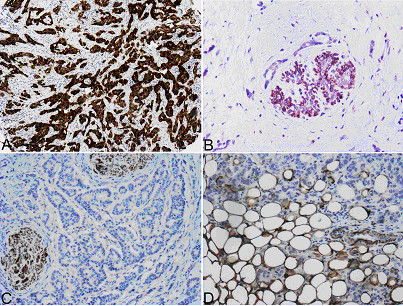
**alphaB-crystallin immunohistochemical detection in cancerous and non-cancerous tissues.** Strong cytoplasmic immunoreactivity of neoplastic cells is shown in **A**, whereas neoplastic cells are negative in **B**-**D**. In normal breast tissue only myoepithelial cells are positive **(B)**. Nerve fibers and adipose tissues are also immunoreactive. (**A**: ×40, **B**-**D**: ×100).

The protein was generally localized in the cytoplasm (Figure 1A), whereas membranous positivity was observed sparsely and it was mainly focal and incomplete. Few cases (n = 46) exhibited nuclear positivity in <5% of the nuclei that was usually not accompanied by cytoplasmic positivity. The nuclear presence of alphaB-crystallin has been previously described [2,46], suggesting a possible role in splicing or in protection of the splicing machinery [46]. Subsequently, tumors were considered as alphaB-crystallin positive based on the cytoplasmic staining of malignant cells. Of note, variable alphaB-crystallin positivity scores were obtained from the examined cores for the same tumor, ranging from negative to strongly positive.Regarding the non-cancerous breast tissue included in histospots, cytoplasmic alphaB-crystallin expression was observed only in myoepithelial cells (Figure 1B). Generally, expression of the protein was not detected in epithelial cells of lobular units or ductal structures. Stromal breast cells were globally negative. Wherever nerves, adipose tissue, vessels (Figure 1C and 1D) and muscle cells could be evaluated, these were consistently positive.

### Association of alphaβ-crystallin with clinopathological features and other markers

The distribution of the examined markers is given in Table 3. The majority of cases were ER- and PgR-positive (74% and 68%, respectively) with high Ki67 (68%). alphaβ-crystallin was expressed in 170 of the 940 breast carcinomas (18.1%). In detail, 770 tumors (81.9%) were negative, 99 (10.5%) were weakly positive and 71 (7.6%) were strongly positive (Table 3). Associations of alphaB-crystallin with clinicopathological parameters are presented in Table 4. High histological grade was more frequent among tumors expressing strong alphaB-crystallin than among tumors with weak or absent staining (p < 0.001). alphaB-crystallin protein expression was not associated with patients’ age, tumor size, histological type or lymph node involvement. The incidence for alphaB-crystallin positive cases (weak or strong expression in the tumors) was by far higher in TNBC (45%) than in non-TNBC (14.5%) patients, while most Luminal A tumors were negative for alphaB-crystallin expression (93.4%, Table 5). Out of the 85 BCP tumors, 44 expressed alphaB-crystallin (52%).

**Table 3:** Distribution of the examined markers

	**N (%)**
**ER (n = 938)**	
Negative	245 (26.1)
Positive	693 (73.9)
**PgR (n = 940)**	
Negative	301 (32.0)
Positive	639 (68.0)
**Ki67 (n = 934)**	
Low	299 (32.0)
High	635 (68.0)
**alphaB-crystallin (n = 940)**	
Negative	770 (81.9)
Weakly positive	99 (10.5)
Strongly positive	71 (7.6)
**BRCA1 (n = 928)**	
Negative	863 (93.0)
Positive	65 (7.0)
***BRCA1*** **(n = 86)**	
WT	78 (90.7)
Mutated	8 (9.3)
**p53 (n = 918)**	
Negative	445 (48.5)
Positive	473 (51.5)
**EGFR (n = 928)**	
Negative	777 (83.7)
Positive	151 (16.3)
**CK5 (n = 923)**	
Negative	802 (86.9)
Positive	121 (13.1)
**CK14 (n = 925)**	
Negative	894 (96.6)
Positive	31 (3.4)
**CK17 (n = 909)**	
Negative	884 (97.2)
Positive	25 (2.8)

WT, wild-type.

**Table 4:** Association of alphaB-crystallin with clinicopathological parameters

	**alphaB-crystallin**			
	**Negative**	**Weakly positive**	**Strongly positive**	**p-value (Pearson chi-square)**
	**N (%)**	**N (%)**	**N (%)**	
Randomization arm				0.556
E-T-CMF	367 (47.7)	55 (55.6)	32 (45.1)	
E-CMF	126 (16.4)	8 (8.1)	13 (18.3)	
ET-CMF	277 (36.0)	36 (36.4)	26 (36.6)	
Age				0.058
<50	301 (39.1)	47 (47.5)	33 (46.5)	
≥50	469 (60.9)	52 (52.5)	38 (53.5)	
Menopausal status				0.402
Premenopausal	351 (45.6)	51 (51.5)	33 (46.5)	
Postmenopausal	419 (54.4)	48 (48.5)	38 (53.5)	
Type of surgery				**<0.001**
MRM	548 (71.2)	53 (53.5)	43 (60.6)	
Breast conserving	222 (28.8)	46 (46.5)	28 (39.4)	
Tumor size (cm)				0.937
≤2	232 (30.1)	35 (35.4)	21 (29.6)	
2.1-5	435 (56.5)	55 (55.6)	40 (56.3)	
>5				
Histological type				0.084
Ductal	586 (76.1)	80 (80.8)	61 (85.9)	
Lobular	89 (11.6)	6 (6.1)	2 (2.8)	
Mixed	59 (7.7)	10 (10.1)	3 (4.2)	
Other	36 (4.7)	3 (3.0)	5 (7.0)	
Number of positive nodes				0.957
0-3	298 (38.7)	36 (36.4)	29 (40.8)	
≥4	472 (61.3)	63 (63.6)	42 (59.2)	
Histological grade				**<0.001**
I-II	406 (52.7)	40 (40.4)	24 (33.8)	
III-Undifferentiated	364 (47.3)	59 (59.6)	47 (66.2)	

N, number; E, epirubicin; T, paxlitaxel; C, cyclophosphamide; M, methotrexate; F, 5-FU; MRM, modified radical mastectomy.

Significant p-values are shown in bold.

**Table 5:** Distribution of examined markers according to subtypes

	**Luminal A**	**Luminal B**	**Luminal-HER2**	**HER2-enriched**	**TNBC**	**BCP**
	**N (%)**	**N (%)**	**N (%)**	**N (%)**	**N (%)**	**N (%)**
**alphaB-crystallin**						
Negative	213 (93.4)	299 (80.6)	109 (83.8)	88 (88.0)	61 (55.0)	41 (48.2)
Weakly positive	8 (3.5)	40 (10.8)	16 (12.3)	11 (11.0)	24 (21.6)	19 (22.4)
Strongly positive	7 (3.1)	32 (8.6)	5 (3.8)	1 (1.0)	26 (23.4)	25 (29.4)
**BRCA1**						
Negative	206 (92.0)	334 (91.0)	125 (97.7)	95 (95.0)	103 (94.5)	79 (92.9)
Positive	18 (8.0)	33 (9.0)	3 (2.3)	5 (5.0)	6 (5.5)	6 (7.1)
***BRCA1***						
WT	19 (90.5)	27 (100.0)	11 (100.0)	10 (100.0)	11 (64.7)	9 (60.0)
Mutated	2 (9.5)	0	0	0	6 (35.3)	6 (40.0)
**p53**						
Negative	148 (67.3)	154 (42.1)	56 (43.8)	41 (42.3)	46 (43.0)	32 (38.6)
Positive	72 (32.7)	212 (57.9)	72 (56.3)	56 (57.7)	61 (57.0)	51 (61.4)

N, number; TNBC, triple-negative breast cancer; BCP, basal core phenotype; WT, wild-type.

alphaB-crystallin expression was significantly more often detected in ER- and PgR-negative tumors, whereas there was a positive association with Ki67, p53, CK5, CK14 and CK17 (Table 6). A strong, positive association was noticed between alphaB-crystallin expression and Ki67, with 81.3% of the cases with positive expression of the former having high expression of the latter. In addition, *BRCA1* mutations were more frequent (p = 0.045) in tumors with strong alphaB-crystallin protein expression compared to tumors with weak or negative expression (33.3% vs. 8.3% vs. 6.2%, respectively). *BRCA1* mutation status was not related to BRCA protein expression (p = 0.40). However, it has to be kept in mind, that DNA for *BRCA1* screening was available for only 86 of the 940 patients included in the present analysis and that this subgroup of patients showed significant differences compared to the overall cohort regarding age, menopausal status, type of surgery, involved axillary lymph nodes and randomization group.

**Table 6:** Association of alphaB-crystallin protein expression with all examined markers

	**alphaB-crystallin**			
	**N (%)**	**N (%)**	**N (%)**	
	**Negative**	**Weakly positive**	**Strongly positive**	**p-value (Pearson chi-square)**
**ER (n = 938)**				<**0.001**
Negative	176 (71.8)	38 (15.5)	31 (12.7)	
Positive	592 (85.4)	61 (8.8)	40 (5.8)	
**PgR (n = 940)**				<**0.001**
Negative	227 (75.4)	38 (12.6)	36 (12.0)	
Positive	543 (85.0)	61 (9.5)	35 (5.5)	
**Ki67 (n = 934)**				<**0.001**
Low	267 (89.3)	17 (5.7)	15 (5.0)	
High	499 (78.6)	81 (12.8)	55 (8.6)	
**BRCA1 (n = 928)**				0.676
Negative	707 (81.9)	91 (10.6)	65 (7.5)	
Positive	52 (80.0)	7 (10.8)	6 (9.2)	
***BRCA1*** **(n = 86)**				**0.045**
WT	61 (78.2)	11 (14.1)	6 (7.7)	
Mutated	4 (50.0)	1 (12.5)	3 (37.5)	
**p53 (n = 918)**				**0.002**
Negative	384 (86.3)	32 (7.2)	29 (6.5)	
Positive	370 (78.2)	66 (14.0)	37 (7.8)	
**EGFR (n = 928)**				<**0.001**
Negative	671 (86.3)	65 (8.4)	41 (5.3)	
Positive	91 (60.3)	32 (21.2)	28 (18.5)	
**CK5 (n = 923)**				**<0.001**
Negative	694 (86.5)	67 (8.4)	41 (5.1)	
Positive	63 (52.1)	31 (25.6)	27 (22.3)	
**CK14 (n = 925)**				<**0.001**
Negative	750 (83.9)	88 (9.8)	56 (6.3)	
Positive	9 (29.0)	10 (32.3)	12 (38.7)	
**CK17 (n = 909)**				<**0.001**
Negative	742 (83.9)	87 (9.9)	55 (6.2)	
Positive	5 (20.0)	9 (36.0)	11 (44)	

WT, wild-type.

Significant p-values are shown in bold.

### Survival analysis

**Figure 2:**
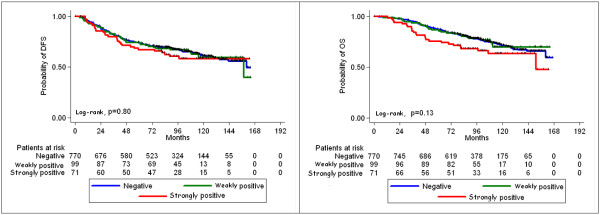
DFS (left) and OS (right) according to alphaB-crystallin protein expression.

After a median follow-up of 105 months (0.1-166.7 months), 5 and 10-year DFS was 73.7% and 60.7%, respectively. Similarly 5 and 10-year OS was 86.4% and 70.8% respectively. In univariate Cox regression analysis (Table 7), *BRCA1* mutation status and alphaB-crystallin, BRCA1 and p53 protein expression were examined regarding their prognostic and predictive value. alphaB-crystallin (3 scale categoric variable) was not associated with either DFS or OS (Table 7 and Figure 2), however, a statistically significant association with OS (but not with DFS) was revealed, when strongly positive tumors were compared against negative and weakly positive tumors in a binary mode (hazard ratio [HR] = 1.52, 95% confidence interval [CI]: 1.01-2.30, Wald’s p = 0.046). Concerning BRCA1 protein expression and *BRCA1* mutational status (Table 7), no significant associations were found with for DFS or OS (Wald’s p-values >0.05 for all cases). Positive p53 tumors exhibited a trend for shorter DFS (HR = 1.21, 95% CI: 0.97-1.50, Wald’s p = 0.088) and shorter OS (HR = 1.24, 95% CI: 0.98-1.65, Wald’s p = 0.066) compared to negative p53 tumors.

**Table 7:** Univariate Cox regression analysis for all examined markers in terms of DFS and OS

		**DFS**		
		**HR**	**95% CI**	**Wald’s p**
**Parameter**	**Category vs. the reference**			
***BRCA1*** **mutation status**	**Mutated**	1.68	(0.59-4.80)	0.33
***BRCA1 protein expression***	**Positive**	0.99	(0.65-1.51)	0.95
**alphaB-crystallin**				0.79
	**Weakly positive**	1.02	(0.72-1.45)	0.91
	**Strongly positive**	1.14	(0.77-1.69)	0.50
**alphaB-crystallin (strongly positive vs. weakly positive/negative)**	**Strongly positive**	1.14	(0.77-1.68)	0.51
**p53 *protein expression***	**Positive**	1.21	(0.97-1.50)	0.088
		**OS**		
		**HR**	**95% CI**	**Wald’s p**
**Parameter**	**Category vs. the reference**			
***BRCA1*** **mutation status**	**Mutated**	1.75	(0.52-5.86)	0.36
***BRCA1 protein expression***	**Positive**	0.95	(0.57-1.58)	0.86
**alphaB-crystallin**				0.14
	**Weakly positive**	0.98	(0.65-1.49)	0.94
	**Strongly positive**	1.52	(1.00-2.31)	0.050
**alphaB-crystallin (strongly positive vs. weakly positive/negative)**	**Strongly positive**	1.52	(1.01-2.30)	0.046
**p53 *protein expression***	**Positive**	1.24	(0.98-1.65)	0.066

HR, hazard ratio; CI, confidence interval.

**Figure 3:**
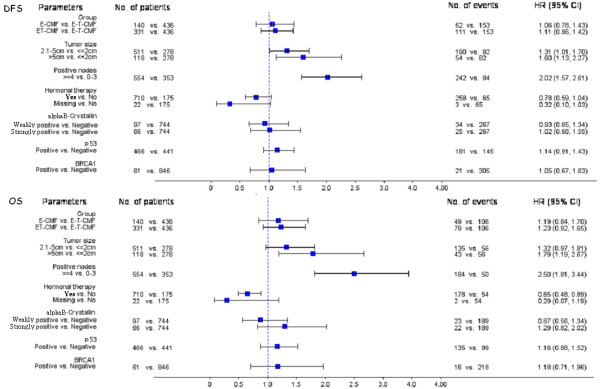
Multivariate Cox regression analysis for DFS and OS presented by forest plots.

There were no significant interactions of alphaB-crystallin with paclitaxel treatment (Wald’s p = 0.72 and p = 0.75 for DFS and OS, respectively). No significant interactions were found either for BRCA1 and p53 protein expression or *BRCA1* mutational status with paclitaxel treatment (Wald test for interaction, all p-values >0.05) (Table 8).In multivariate Cox regression analysis, alphaB-crystallin, BRCA1 and p53 protein expression were not prognostic for DFS and OS. Among clinical characteristics, tumor size of more than 5 cm and ≥4 positive axillary nodes were independent prognostic factors and were associated with poor DFS and OS (Figure 3).

**Table 8:** Interaction of all examined markers with paclitaxel treatment in terms of DFS and OS

	**DFS**			**OS**		
	**HR**	**95% CI**	**Wald’s p**	**HR**	**95% CI**	**Wald’s p**
alphaB-crystallin * Treatment			*0.72*			*0.75*
alphaB-crystallin neg vs. pos in non-paclitaxel treated	1.21	0.48-3.01		0.88	0.35-2.23	
alphaB-crystallin neg vs. pos in paclitaxel treated	0.81	0.53-1.24		0.60	0.38-0.97	
Non-paclitaxel treated vs. paclitaxel treated in alphaB-crystallin neg	1.23	0.92-1.63		1.35	0.97-1.86	
Non-paclitaxel treated vs. paclitaxel treated in alphaB-crystallin pos	0.82	0.31-2.17		0.93	0.35-2.47	
BRCA1 * Treatment			*0.55*			*0.99*
BRCA1 neg vs. pos in non-paclitaxel treated	1.43	0.34-5.91		1.01	0.24-4.20	
BRCA1 neg vs. pos in paclitaxel treated	0.96	0.61-1.49		1.02	0.59-1.77	
Non-paclitaxel treated vs. paclitaxel treated in BRCA1 neg	1.19	0.90-1.58		1.29	0.94-1.78	
Non-paclitaxel treated vs. paclitaxel treated in BRCA1 pos	0.79	0.18-3.43		1.31	0.29-5.85	
***BRCA1*** * Treatment			*0.37*			*0.48*
*BRCA1* WT vs. mut in non-paclitaxel treated^1^	.	.		.	.	
*BRCA1* WT vs. mut in paclitaxel treated	0.61	0.21-1.76		0.64	0.19-2.17	
Non-paclitaxel treated vs. paclitaxel treated in *BRCA1* WT	0.76	0.25-2.25		0.29	0.06-1.37	
Non-paclitaxel treated vs. paclitaxel treated in *BRCA1* mut^1^	.	.		.	.	
p53 * Treatment			*0.35*			*0.58*
p53 neg vs. pos in non-paclitaxel treated	1.06	0.61-1.84		0.94	0.50-1.78	
p53 neg vs. pos in paclitaxel treated	0.80	0.63-1.02		0.77	0.58-1.03	
Non-paclitaxel treated vs. paclitaxel treated in p53 neg	1.36	0.82-2.23		1.37	0.76-2.47	
Non-paclitaxel treated vs. paclitaxel treated in p53 pos	1.02	0.72-1.43		1.13	0.77-1.65	

*: interaction; ^1^: There were no patients with *BRCA1* mutations in the non-paclitaxel treated group.

HR, hazard ratio; CI, confidence interval; pos, positive; neg, negative; mut, mutated; WT, wild-type.

## Discussion

In this study, including a large cohort of breast cancer cases, it is demonstrated that alphaB-crystallin is expressed in a low percentage of breast carcinomas (18,1%), which drops to 7.6% when the Moyano-suggested cut off for strong positivity (>30% positive cells) is applied. These findings are within the reported range of alphaB-crystallin protein expression (10 and 17%) [7,18,19]. However, two studies reported high rates of alphaB-crystallin expression with the same antibody (64.6 and 88%) [11,16]. Differences in TMA construction and IHC evaluation may account for this discrepancy. In both studies, alphaB-crystallin was scored according to the highest intensity even in a few cells regardless of how many cells actually expressed this protein. In addition, 81 of the 82 cases [11] were TNBC, which often express alphaB-crystallin; a high percentage of TNBC can also be inferred from the ER/PgR/HER2 profiles of the tumors examined [16].

It seems that BCP express this protein more often. As described here, 45% (50/111) of TNBC and almost half of BCP were alphaB-crystallin positive. Sitterding et al. suggested that alphaB-crystallin is a sensitive and specific marker for BCP [29]. In addition, it is also mentioned that alphaB-crystallin expression is related to basal markers, such as CK5, CK14 and CK17. Considering that alphaB-crystallin is expressed in normal myoepithelial cells it can be viewed as a basal/myoepithelial marker or as the organizer of stratified cytokeratins through its ability to regulate the cytoskeleton organization [47]. The fact that almost half of BCP, as shown in this study, express alphaB-crystallin raises the question why the remaining BCP cases fail to express this protein. This finding could be due to immunostain heterogeneity or BCP heterogeneity as they represent a heterogeneous group. The effort of identifying new molecular markers to subdivide BCP subtypes is still ongoing [22,48]. On the other hand, the heterogeneous pattern of alphaB-crystallin expression throughout the tumor area, as observed in our study, prompts for caution when using TMAs for the assessment of tumor immunoreactivity of this marker.

The oncogenic role of alphaB-crystallin has been demonstrated in two human mammary epithelial cell lines and in experimental mice models, where it results in the development of invasive mammary carcinomas [7]. Recently, it has been suggested that alphaB-crystallin promotes tumor progression by enhancing endothelial cell survival, resulting in efficient tumor vascularization [49,50]. There are several studies that introduce sHsps and especially Hsp27 and alphaB-crystallin as contributors to the epithelial-mesenchymal transition (EMT) process. Both proteins interact with the cytoskeleton and regulate its dynamic status by inducing mesenchymal-like spindle cells; thus, they may promote cancer cell invasion and metastasis [47,51]. Consequently, alphaB-crystallin may contribute to an aggressive behavior of tumors; this is in line with our observation that alphaB-crystallin is more commonly found in BCP and in those non-TNBC that have a high histological grade and proliferation rate. Of note, in the current study the majority of non-TNBC alphaB-crystallin positive cases were Luminal B tumors, which by definition have a high Ki67 labeling index. The expression of alphaB-crystallin in a subset of non-TNBC has been mentioned by other studies, as well [7,11,18].

BCP constitute a tumor subgroup associated with *BRCA1* mutations. Tumors of patients with *BRCA1* germline mutations usually display the basal core phenotype [52,53]. In this study we found that alphaB-crystallin is associated with BCP and *BRCA1* mutational status but not with BRCA1 protein expression. Moreover, no significant association was found between mutational status and protein expression. Despite the small sample size (n = 8) of *BRCA1* mutant cases, this result is in line with the global view that IHC does not reliably reflect *BRCA1* gene status and cannot be used for assessing the impact of BRCA1 protein expression on prognosis [52,54-56]. We also found a strong association between alphaB-crystallin and p53 expression, which is again in line with BCP – *BRCA1* mutant tumors. alphaB-crystallin overexpression prevents apoptosis, through the interaction of the p53 down regulated genes, such as bax or pro-caspase3 [57,58]. Recently, it was shown that alphaB-crystallin binds to p53 to sequester its translocation to the mitochondria during hydrogen peroxide induced apoptosis [58]. Hence, like other Hsps, alphaB-crystallin interacts with p53 and modulates its function. On the other hand, it is believed that p53 is involved in the regulation of Hsps in cancer and p53 mutations result in an increase of Hsp transcripts [59,60]. Our IHC findings further support these interactions, specifically between the alphaB-crystallin and p53 proteins.

alphaB-crystallin expression has been associated with poor clinical outcome in breast [7,11], head and neck [17] and hepatocellular carcinoma [61]. Moyano et al. found that this biomarker predicts for shorter disease-specific survival, independent of other prognostic markers. By contrast, Chelouche-Lev et al. reported that this sHsp inadequately predicts patient outcome [16], despite the fact that it is strongly associated with the presence of lymph node metastasis. Herein we observed a statistically significant association between strong protein expression of alphaB-crystallin (as determined by Moyano) and overall survival. This may indicate that alphaB-crystallin overexpression actually contributes to tumor aggressiveness that has a negative impact on patients’ survival. It should be noted that only 7.6% (71/940) of the patients were strongly positive, when using the above cut-off for overexpression, which might have produced biased results. Nevertheless, the present multivariate analysis data indicated that alphaB-crystallin might not be considered to be an independent prognostic marker in breast cancer.

Regarding to its predictive role, increased expression of alphaB-crystallin has been associated with acquired resistance to cisplatin, etoposide and fotemustine [62]. Ivanov et al. described that there is an association between alphaB-crystallin expression and resistance to neoadjuvant chemotherapy in breast cancer, suggesting its possible role in the identification of a chemoresistant subset of TNBC [18]. In this particular study alphaB-crystallin was not shown to be a predictive marker for response to paclitaxel therapy. Similarly, in the present study, we did not find any interaction between alphaB-crystallin and taxane-containing regimens. Although this is a negative result, to our knowledge, this is the first report attempting to establish an interaction between taxane-based therapies and alphaB-crystallin protein expression.

## Conclusions

alphaB-crystallin cannot be considered to be a marker for BCP but a protein expressed in carcinomas with aggressive biologic nature that are characterized by high labeling index (Ki67/mib1), triple-negative phenotype, basal protein expression, p53 overexpression and high histological grade. However, alphaB-crystallin does not seem to have an independent impact on patient prognosis. Evidently, since results on outcome are IHC cut-off sensitive, the applied cut-off needs further validation. Lastly, although alphaB-crystallin protein expression was not shown to be a predictive marker for taxane-based therapy, to our knowledge this is the first study to evaluate the association between alphaB-crystallin and taxane-based therapy in a large cohort of patients. Further studies are needed to evaluate this result in a balanced patient population.

## Abbreviations

TNBC: Triple-negative breast carcinoma; sHsps: Small heat shock proteins; BLBC: Basal-like breast carcinomas; E: Epirubicin; T: Paxlitaxel; C: Cyclophosphamide; M: Methotrexate; F: 5-FU; FFPE: Formalin-fixed paraffin-embedded; TMAs: Tissue microarrays; IHC: Immunohistochemistry; BCP: Basal core phenotype; FISH: Fluorescence in situ hybridization; JT test: Jonckheere-Terpstra trend test; DFS: Disease-free survival; OS: Overall survival; ΜRΜ: Modified radical mastectomy; HR: Hazard ratio; CI: Confidence interval; EMT: Epithelial-mesenchymal transition.

## Competing interests

The authors declare that they have no competing interests.

## Authors’ contributions

TK conceived of the study, participated in its design as well as in the acquisition, analysis and interpretation of data and drafted the manuscript. FS conceived of the study, participated in its design as well as in the analysis and interpretation of data and drafted the manuscript. MB conceived of the study, participated in its design as well as in the acquisition, analysis and interpretation of data and drafted the manuscript. IK participated in the acquisition, analysis and interpretation of data and revised the manuscript critically. VK participated in the interpretation of data, drafted the manuscript and revised it critically. AGE performed the statistical analysis. IK participated in the acquisition, analysis and interpretation of data, drafted the manuscript and revised it critically. CP participated in the acquisition of data and revised critically the manuscript. AB participated in the analysis and interpretation of data. HG participated in the acquisition of data and revised critically the manuscript. AK contributed in the acquisition of data. DVS contributed in the acquisition of data. GP participated in the acquisition of data and revised critically the manuscript. IE participated in the acquisition, analysis and interpretation of data. DP contributed in the acquisition of data. GF conceived of the study, participated in its design as well as in the analysis and interpretation of data, drafted the manuscript and revised it critically. All authors read and approved the final manuscript.

## Pre-publication history

The pre-publication history for this paper can be accessed here:

http://www.biomedcentral.com/1472-6890/14/28/prepub
